# What is the actual prevalence of migraine?

**DOI:** 10.1002/brb3.950

**Published:** 2018-05-02

**Authors:** Wei Z. Yeh, Leigh Blizzard, Bruce V. Taylor

**Affiliations:** ^1^ Neurology Department Royal Hobart Hospital Hobart Tas. Australia; ^2^ Menzies Institute for Medical Research University of Tasmania Hobart Tas. Australia

**Keywords:** epidemiology, genetics, migraine, neurology, pain, prevalence

## Abstract

**Objectives:**

Population prevalence studies of migraine report prevalence rates of between 2.6 and 21.7%, with an average of ~12%. However, migraine prevalence among neurologists is reported to be significantly higher, between 27.6% and 48.6%. Increasing knowledge of the protean manifestations of migraine may explain this difference. Similarly, under‐recognition of migraine in control groups may explain the lack of genetic and biomarker findings in this disorder. We therefore sought to determine the prevalence of migraine in an admixed group of individuals with varied knowledge of migraine symptomatology.

**Methods:**

Attendees at the Australian and New Zealand Association of Neurologists Annual Scientific Meeting (ANZAN ASM) 2017 were surveyed anonymously. Those surveyed included three groups: neurologists, neurology trainees, and others including nonclinical researchers, members of lay organizations, and representatives of the pharmaceutical industry.

**Results:**

In total, 313 of 606 attendees responded (51.7%). 65.9% of neurologist, 57.4% of trainee, and 52.5% of others respondents had a personal history of migraine, with the difference between neurologists and others being statistically significant (*p* = .03). Migraine in migraineurs and nonmigraine headache in nonmigraineurs were nearly all self‐diagnosed. Among neurologist migraineurs, 51.2% experienced migraine with aura and 43% migraine without aura.

**Conclusions:**

Migraine prevalence is significantly higher in neurologists compared to non‐neurologists and at least 2–3 times higher than reported in population prevalence studies. This may be due to significant under‐recognition of migraine in non‐neurologists. This under‐recognition of migraine may significantly influence the search for genetic predictors and biomarkers of migraine.

## 1 INTRODUCTION

Migraine is a common primary, although clearly not exclusively, headache disorder characterized by recurrent episodes of headache often associated with nausea, vomiting, photophobia, and phonophobia. The manifestations of migraine clinically can be protean and may not include headache or only mild headache (although this is a primary requirement for the diagnosis in many classifications; Headache Classification Committee of the International Headache Society (IHS), 2013). It is a major cause of disability and is among the top 10 causes of years lived with disability in the world (Global Burden of Disease Study 2013 Collaborators, 2015).

Migraine prevalence is reported as between 2.6% and 21.7%, with an average of ~12%, with variation between countries and also between studies within the same country (Burch, Loder, Loder, & Smitherman, 2015; Lipton, Stewart, Diamond, Diamond, & Reed, 2001; Merikangas, 2013; Wang, 2003). There is a strong familial link in migraineurs suggesting a large genetic component to migraine risk (Dzoljic et al., 2014; Hernandez‐Latorre & Roig, 2000; Merikangas, 2013; Russell & Olesen, 1995; Stewart, Staffa, Lipton, & Ottman, 1997). Twin studies have shown that migraine is a complex genetic disease with interplay between genetic and environmental factors, with heritability estimated to be as high as 65% (Gervil, Ulrich, Kaprio, Olesen, & Russell, 1999; Honkasalo et al., 1995; Mulder et al., 2003; Svensson, Larsson, Waldenlind, & Pedersen, 2003; Ulrich, Gervil, Kyvik, Olesen, & Russell, 1999).

Surprisingly, despite several large genomewide association studies (GWAS) being undertaken (Anttila et al., 2010; Chasman et al., 2011; Freilinger et al., 2012), no conclusive candidate genes have been identified, with a recent systematic re‐evaluation of 27 candidate genes identifying none as being statistically significant (De Vries et al., 2016).

Migraine prevalence in neurologists is higher compared to the general population with prevalence of up to 48.6% seen among neurologists (Alstadhaug, Hernandez, Naess, & Stovner, 2012; Donnet, Becker, Allaf, & Lantéri‐Minet, 2010; Evans & Evans, 2010; Evans & Ghosh, 2016; Evans, Lipton, & Ritz, 2007; Evans, Lipton, & Silberstein, 2003; Gil‐Gouveia, 2014; Lu, Wang, & Fuh, 2006). The reason for this is almost certainly better self‐recognition of symptoms corresponding to migraine by those trained and experienced in the manifestations of migraine. This was shown in a study in which only just over half of individuals identified as having a diagnosis of migraine recognized that their headache was in fact migraine (Lipton, Stewart, & Liberman, 2002).

In this study, we aim to examine migraine prevalence among neurologists, neurology trainees, and non‐neurologists/trainees at a national neurology meeting. We hypothesize that the neurologists will have the highest prevalence, followed by trainees and then non‐neurologists/trainees, and that the prevalence of migraine among neurologists will be significantly higher than the reported population level prevalence of migraine.

If our hypothesis is correct, this may explain the lack of genetic associations and biomarkers found due to under‐recognition within population studies and therefore inclusion of significant numbers of false negatives in the control groups. If as hypothesized recognition is the problem, then careful phenotyping of the control and affected study groups is the only alternative in the absence of a biomarker of disease.

## 2 METHODS

### 2.1 Subjects

Attendees at the Australian and New Zealand Association of Neurologists Annual Scientific Meeting (ANZAN ASM) 2017 were surveyed anonymously. Attendees were considered in three groups: (1) neurologists, (2) neurology trainees, and (3) others, with this group consisting predominantly of representatives of pharmaceutical, scientific and lay support organizations and nonclinical research personnel. Ethical approval was obtained from the Southern Tasmanian human research ethics committee.

### 2.2 Survey

A single‐page survey was designed with questions divided into three main sections. The first section asked demographical information including gender, age, and group. The second section enquired regarding family history of migraine, personal history of migraine or headache, and who made the diagnosis of the headache phenotype. The third section was directed at neurologists and asked whether they considered themselves a headache specialist, whether their history of migraine influenced their career decision, and what migraine types were experienced. A description of study objectives and the International Classification of Headache Disorders (ICHD) third edition criteria for migraine were provided.

### 2.3 Statistical analysis

The mean and standard deviation (*SD*) of each continuous variable, and the percentage and frequency of the attributes of each categorical variable, are reported to summarize the characteristics of each group of participants. Tests of the statistical significance of differences between groups were undertaken using analysis of variance methods (continuous variables) and chi‐square tests of independence (categorical variables). Prevalence and ratios of prevalence were estimated using generalized linear models with a binomial error distribution and logarithmic link (log binomial regression). By adding covariates for age and sex to the model, the estimated prevalence and prevalence ratios were adjusted for those factors. A two‐sided *p* value < .05 was considered statistically significant. Statistical analyses were performed in Microsoft Excel and using Stata (Stata Corporation).

## 3 RESULTS

Of 606 total attendees at ANZAN ASM 2017, 313 completed the survey for an overall response rate of 51.7%. One respondent did not answer which group they belonged to and was excluded from analysis. Respondent demographics and proportions with family and personal history of migraine are given in Table 1. The overall registration figure included 254 ANZAN members (neurologists/trainees) giving a response rate of 76% for these two groups (we cannot subdivide further due to lack of data).

**Table 1:** Respondent characteristics

	Neurologists (*N* = 138)	Neurology trainees (*N* = 54)	Others (*N* = 120)	All (*N* = 312)
Gender				
Female (%)	50 (36.2)	24 (44.4)	70 (58.3)	144 (46.2)
Male (%)	88 (63.8)	30 (55.6)	50 (41.7)	168 (53.8)
Age—years				
Mean (*SD*)	49.4 (11.7)	32.3 (4.1)	43 (10.1)	44 (11.8)
Range	30–82	27–45	22–75	22–82
Migraine				
Family history (%)	76 (55.1)	18 (33.3)	49 (40.8)	143 (45.8)
Personal history (%)	91 (65.9)	31 (57.4)	63 (52.5)	185 (59.3)

In terms of personal history of migraine, neurologists had the highest proportion (65.9%), followed by neurology trainees (57.4%), and others (52.5%). The difference between neurologists and others was statistically significant (proportion difference 13.4%, 95% confidence interval (CI) 1.51%–25.4%, *p* = .03) but the differences between neurologists and trainees, and trainees and others, were not (*p* = .27 and 0.55, respectively).

A family history of migraine was highest again in neurologists (55.1%). The differences between neurologists and trainees (proportion difference 21.7%, 95% CI 6.67%–36.8%, *p* = .007), and neurologists and others (proportion difference 14.2%, 95% CI 2.15%–26.3%, *p* = .02) were significant, but not between trainees and others (*p* = .35).

Examining prevalence by sex, neurologists had a higher prevalence of migraine compared to others with this being significant for males (*p* = .003) but not for females (*p* = .37; Table 2). Within groups, there was no significant difference between males and females except for migraine prevalence in others (*p* = .002).

**Table 2:** Personal history and family history of migraine by sex

	Neurologists (*N* = 138)	Trainees (*N* = 54)	Others (*N* = 120)	All (*N* = 312)	*p*‐Values^a^
Female—*n*	50	24	70	144	
Personal history (%)	36 (72)	11 (45.8)	45 (64.3)	92 (63.9)	.03 .37 .11
Family history (%)	28 (56)	9 (37.5)	31 (44.3)	68 (47.2)	.14 .21 .56
Male—*n*	88	30	50	168	
Personal history (%)	55 (62.5)	20 (66.7)	18 (36)	93 (55.4)	.68 .003 .008
Family history (%)	48 (54.5)	9 (30)	18 (36)	75 (44.6)	.02 .04 .58

a *p*‐Values comparing neurologists to trainees, neurologists to others, and trainees to others, respectively.

After adjustment for age and sex, the prevalence of a personal history of migraine among trainees was statistically indistinguishable from prevalence among the neurologists (prevalence ratio PR = 0.91, *p* = .56), but prevalence among others was significantly lower (PR = 0.80, *p* = .03) than the prevalence among neurologists. In this model, age (PR = 1.01 per year, *p* = .04) and female sex (PR = 1.19, *p* = .05) were significant predictors of the prevalence of a personal history of migraine.

### 3.1 Diagnosis of migraine and headache

In those with a personal history of migraine, the majority self‐diagnosed their migraine (85.7% in the neurologist group, 90.3% in the trainee group, and 44.4% in the others group). 33.3% of migraineurs in the others group were diagnosed by a non‐neurologist physician, followed by 15.9% by neurologists.

Of the nonmigraineurs, 36.2% in the neurologist group, 60.9% in the trainee group, and 40.4% in the others group described having previous headaches. All of the respondents in the neurologist and trainee groups had self‐diagnosed their headaches as not migraine, compared to 91.3% in the others group. The remaining 8.7% in the others group were diagnosed by non‐neurologist physicians.

Thirteen female neurologists and 15 male neurologists described themselves as headache specialists. Female nonheadache specialists had the highest proportion with a personal history of migraine, followed by female headache specialists, male nonheadache specialists, and male headache specialists (73% vs. 69.2% vs. 64.4% vs. 53.3%, P = NS).

**Figure 1:**
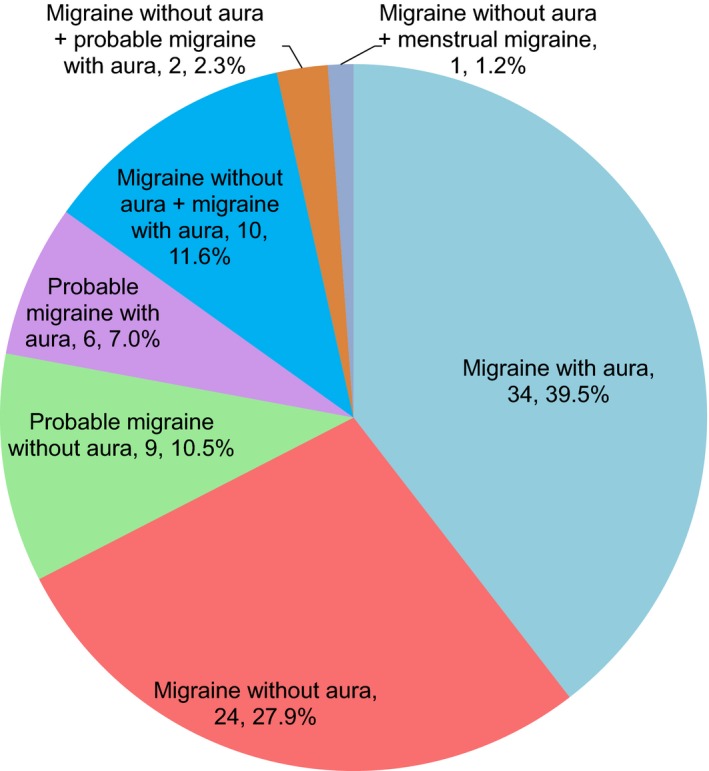
Neurologists with a personal history of migraine and their migraine types. Labels formatted as group, frequency, proportion as percent. Migraine with aura, migraine without aura, and probable migraine with aura values, and percentages refer to respondents who listed these alone

Of the neurologists with a personal history of migraine, two (2.2%) responded that this influenced their career decision to become a neurologist. 86 provided responses regarding their migraine type (Figure 1), with the most common types being migraine with aura (51.2%) and migraine without aura (43.0%). Thirteen responded that they had experienced two migraine types. In terms of aura subtype in respondents with migraine with aura and probable migraine with aura, the most common was typical aura (86.7%) followed by retinal migraine (8.9%) and brainstem aura (4.4%). No other aura subtypes were selected.

## 4 DISCUSSION

To our knowledge, this is the first study examining migraine prevalence in neurologists to also include non‐neurologist and nonphysician groups for comparison. Our overall response rate of 51.7% was comparable to other survey‐based studies of migraine in neurologists, although our response rate from neurologists and trainees of 76% is higher compared to most of these previous studies (Alstadhaug et al., 2012; Donnet et al., 2010; Evans & Evans, 2010; Evans & Ghosh, 2016; Evans et al., 2003, 2007; Gil‐Gouveia, 2014; Lu et al., 2006). As in previous studies, we demonstrated a significantly higher prevalence of migraine in neurologists (65.9%) than would be expected based on population prevalence studies. Groups demonstrated increasing prevalence with neurological experience, with the highest prevalence in neurologists and lowest prevalence in others. The proportion with a positive family history of migraine was also higher in neurologists compared to others. Better recognition and increased awareness of migraine and its features among neurologists are highly likely to explain these differences.

Stratifying by sex also demonstrated higher prevalence of migraine and family history of migraine in neurologists compared to non‐neurologists/trainees. The differences were more marked in males than females. This may be due to lower awareness of migraine in males compared to females, and thus, population prevalence of migraine in males may be underestimated.

Nearly all of the diagnoses of migraine in migraineurs and nonmigraine headache in nonmigraineurs were made by the respondent. Among nonmigraineurs with headache in the others group, none were diagnosed by a neurologist. This highlights the significant risk of under‐recognition and undertreatment of migraine in this group and by inference the general population.

Apart from increased recognition, another explanation for higher migraine prevalence in neurologists is that their history of migraine predisposes them to pursue a career in neurology. This hypothesis was not supported by our findings, with only 2 of 91 neurologists with a history of migraine responding that their migraine history influenced their career decision.

The most common type of migraine experienced by the neurologist group was migraine with aura. This is again different to population studies where migraine without aura is more common than migraine with aura. Our finding may again be due to better recognition of aura by neurologists compared to the general population, with this also supported by findings from a study of Norwegian neurologists (Alstadhaug et al., 2012). The presence of aura was based on neurologists' self‐assessment, and another possible reason for our finding is varied definitions of what constitutes aura among neurologists as compared to strict definition as per ICHD criteria.

The proportion with a personal history of migraine in the others group is higher than might be expected in the general population. This group mostly consisted of representatives from pharmaceutical and scientific companies, and thus, they were likely to have greater education levels regarding neurological diseases relative to the population average, with increased knowledge and therefore better recognition of migraine. There is, however, also likely significant selection bias particularly of the others group with migraineurs more likely to participate in our study. There may also be significant differences in terms of demographics between responders and nonresponders. It could be expected that if a larger proportion of non‐neurologists/trainees were captured, the difference in migraine prevalence between neurologists and others may be larger than demonstrated in our study. The lower number of respondents in the neurology trainee group reflects lower overall numbers in this group, given there are only 130 trainees in Australia and New Zealand. On adjustment for age and sex, this group was statistically similar to the neurologist group.

Our estimated overall response rate was 51.7% which may introduce response bias, with those with a personal history of migraine potentially more likely to respond to a migraine prevalence study. The response rate in neurologists/trainees was 76% which minimizes response bias of these groups. However, even assuming the maximum denominator possible for the neurologist and trainee groups, the migraine prevalence would still be significantly higher (twofold to threefold) than expected based on population prevalence studies. It is highly likely that the responses from the neurologist/trainee groups captured the whole spectrum of migraine with several respondents noting that they had only ever had a handful of migraines in their lives and with several also noting that they had only ever had migraine auras. Our study relied on self‐reporting of migraine with the assumption that neurologists and trainees were familiar with ICHD criteria for migraine and its subtypes, although a copy of these criteria was provided to participants. However, it cannot be excluded that some participants, particularly others, were not familiar with these criteria and thus there may be both under‐ and over‐recognition of migraine.

Our findings show a significantly higher prevalence of migraine in neurologists compared to non‐neurologists. However, given the limitations of our study, conclusions should be drawn with caution. The most likely explanation is under‐recognition of migraine among non‐neurologists and increased awareness of migraine and its manifestations by neurologists. Thus, the true population prevalence of migraine may be significantly higher than previously determined and may explain the difficulties in finding genes for migraine. Many clinic‐ and population‐based migraine GWAS have unscreened population‐matched controls inferring that these control groups are expected to have similar migraine prevalence to the general population (Anttila et al., 2010; De Vries et al., 2016). Questionnaires are often used to select for cases and controls in population‐based GWAS thus introducing the possibility of misrecognition of nonmigraineur status among controls. Careful selection and phenotyping of control and case groups in migraine genetic, biomarker, and other studies may be the only methodology to allow for true differences to be identified.

## CONFLICTS OF INTEREST

None declared.
